# Case Report: Metastasis of low-grade endometrial stromal sarcoma to the inferior vena cava and right atrium: a case of successful one-stage surgical resection with favorable early outcome

**DOI:** 10.3389/fradi.2025.1723859

**Published:** 2026-01-07

**Authors:** Jinglian Tu, Xiaopei Xu, Fengbo Huang

**Affiliations:** 1 Department of Radiology, Second Affiliated Hospital, Zhejiang University School of Medicine, Hangzhou, China; 2 Department of Pathology, Second Affiliated Hospital, Zhejiang University School of Medicine, Hangzhou, China

**Keywords:** cardiac metastasis, CT, heart tumor, intraoperative transesophageal echocardiography, intravascular extension, low-grade endometrial sarcoma (LGESS), MRI, right atrial mass

## Abstract

Low-grade endometrial stromal sarcoma (LGESS) is a rare uterine malignancy; metastasis to the inferior vena cava (IVC) and right atrium is exceptionally rare and presents significant diagnostic and therapeutic challenges. We report the case of a 37-year-old woman presenting with progressive abdominal mass enlargement, palpitations, and dyspnea. She had undergone a hysteroscopic resection for presumed uterine myoma one year prior, which was subsequently re-evaluated as LGESS. Multimodal imaging comprising ^18^F-FDG PET/CT, MRI, CT, and echocardiography was implemented for systemic staging and hemodynamic assessment, then revealed a solid uterine mass involving the adnexa (FIGO Stage IVB) and identified hypermetabolic tumor thrombi extending from the IVC into the right atrium and pulmonary arteries. A coordinated one-stage radical resection was performed, involving total hysterectomy and removal of intracardiac thrombi under cardiopulmonary bypass. Postoperative pathology and immunohistochemistry confirmed LGESS (CD10+, ER+, PR+) with extensive lymphovascular invasion. The patient recovered uneventfully with no residual disease on follow-up and commenced adjuvant letrozole therapy. This case highlights the necessity of multimodal imaging for accurate staging of complex vascular involvement and demonstrates that aggressive one-stage surgical management is a viable strategy to achieve locoregional control and favorable early outcomes for advanced LGESS with cardiac metastasis.

## 1 Introduction

Low-grade endometrial stromal sarcoma (LGESS) is a rare malignancy arising from the mesenchymal tissue of the endometrium. Characterized by a low mitotic rate and indolent behavior, LGESS often presents diagnostic challenges, particularly when metastasis occurs to unusual sites. The clinical manifestation is typically nonspecific, with symptoms such as abnormal uterine bleeding or pelvic pain, which can delay diagnosis until the disease is advanced. While LGESS primarily remains confined to the uterus, distant metastasis can occur, most frequently to the lungs, liver, and peritoneum (1, 2). Metastasis to the inferior vena cava (IVC) and right atrium is exceptionally rare, complicating both diagnosis and treatment. These sites of metastatic involvement can lead to severe hemodynamic consequences, such as venous obstruction and embolic events, necessitating a multidisciplinary therapeutic approach. Surgical resection, though the cornerstone of treatment, becomes particularly complex when the tumor involves critical vascular structures (3).

Timely diagnosis and intervention are essential for optimizing outcomes in LGESS, especially given its propensity for late metastasis. Early detection allows for prompt surgical intervention, which remains the mainstay of treatment. Due to the indolent nature of the disease, LGESS may remain asymptomatic until it reaches an advanced stage, underscoring the importance of vigilant surveillance in at-risk populations (4). Advanced imaging modalities, including ultrasound, computed tomography (CT), magnetic resonance imaging (MRI), and positron emission tomography-computed tomography (PET-CT), play a critical role in identifying metastatic spread, particularly to atypical sites such as the IVC and right atrium. In particular, molecular imaging has demonstrated significant value in the depiction of rare metastatic sites that may be overlooked by conventional anatomical imaging. For instance, ^18^F-FDG PET (5) has proven effective in identifying atypical metastases through metabolic characterization, while SPECT (6) imaging offers high diagnostic utility in detecting rare skeletal metastases in gynecological malignancies, further reducing the risk of under-staging. These imaging techniques are invaluable for assessing the extent of the disease, determining vascular involvement, and guiding therapeutic decision-making, which may include preoperative embolization or vascular stenting (4, 7, 8).

Metastasis of LGESS to both the IVC and right atrium is an exceedingly rare phenomenon, with few cases documented in the literature (7, 9–11). The importance of early intervention in preventing extensive metastasis has been emphasized by several studies (2, 12, 13), however, metastasis to the heart remains an understudied and poorly understood aspect of the disease. In this context, our case is noteworthy for the presence of cancerous emboli extending from the IVC to the right atrium, representing a significant clinical challenge. Previous reports, including those by Jacki et al. (14) and Gnangnon (15), have described distant metastases but not direct right atrial involvement, highlighting the rarity of this presentation. Moreover, studies such as those by Oliva et al. (16) have demonstrated that survival outcomes in metastatic LGESS are closely tied to the extent of disease and the timing of surgical intervention. The current case, which involved a one-stage surgical resection of both the primary tumor and metastases to the IVC and heart, underscores the potential of coordinated multidisciplinary approaches to managing such complex cases and provides valuable insights into future treatment strategies.

## 2 Case presentation

### 2.1 Patient information

A 37-year-old woman was referred to our hospital after presenting with a palpable mass in the lower abdomen that had been present for over a year. In December 2023, she underwent a hysteroscopic resection of a uterine mass at a previous institution under the diagnosis of uterine myoma. However, postoperative pathological examination revealed the mass to be a LGESS. Postoperative enhanced CT scans from the referring institution suggested the possibility of tumor metastasis, prompting a recommendation for further surgical intervention, specifically a total hysterectomy. The patient, however, did not pursue this recommended treatment.

Two months prior to her presentation at our hospital, she was hospitalized at another facility for a medical abortion. A follow-up CT scan during this admission revealed multiple uterine masses, which were suspected to be malignant, with metastasis to both ovaries. Additionally, tumor thrombi were identified in the left ovarian vein, left renal vein, bilateral iliac veins, and IVC, along with a suspected metastasis to the left edge of the liver. Over the past two months, the patient had developed palpitations at rest, occasional dyspnea, and a noticeable increase in the size of the abdominal mass. These worsening symptoms led to her admission to our hospital in September 2024.

The patient had a history of one childbirth (para 1) and previously regular menstrual cycles. Her past medical history was notable for a hysteroscopic resection of a uterine polyp in 2019, which was pathologically confirmed as benign. She denied any history of smoking or alcohol consumption and any significant surgical or traumatic history. There was no family history of hereditary diseases or malignancies. Upon admission in September 2024, she reported prolonged and heavy menstrual bleeding which had started on September 10, 2024, and persisted to the present.

### 2.2 Clinical findings and diagnosis

Upon admission, routine laboratory tests revealed the following findings: red blood cells were 2.77 × 10^12^/L, hemoglobin was 80 g/L, platelets were 124 × 10^9^/L, and D-dimer was 1550 µg/L (FEU). Tumor markers showed human epididymis protein 4 (HE4) at 136.28 pmol/L, while urinalysis demonstrated occult blood at 3+ and urinary red blood cells at 314/µL.

**Figure 1:**
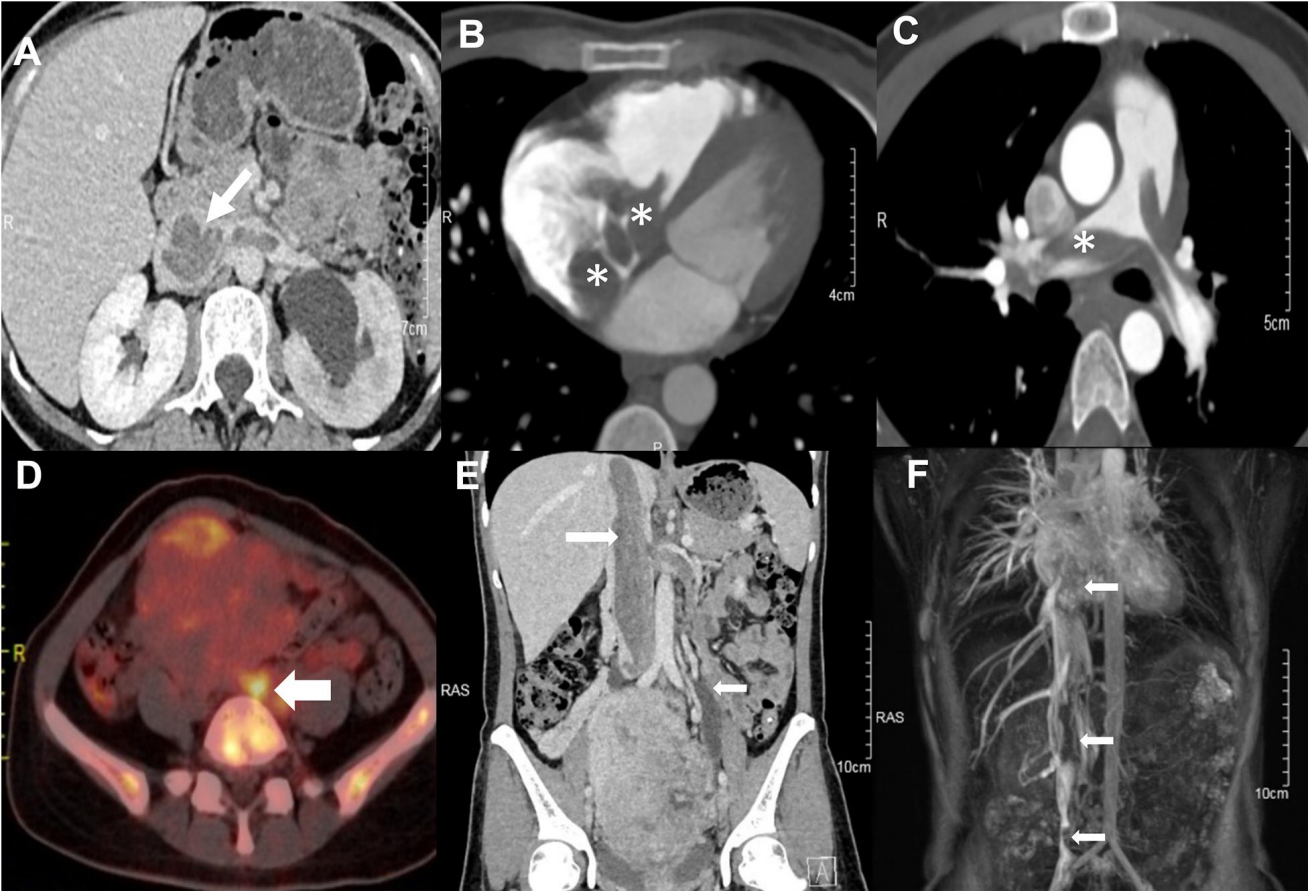
Preoperative imaging findings. **(A)** Axial view showing the presence of tumor thrombus in the left renal artery and extending into the inferior vena cava. **(B)** Axial view revealing the tumor thrombus extending from the right atrium through the tricuspid valve into the right ventricle. **(C)** Axial view demonstrating tumor thrombus within the main pulmonary artery as well as the left and right pulmonary arteries. **(D)** Fused PET/CT image showing the hypermetabolic tumor thrombus in the inferior vena cava and the left ovarian vein. **(E)** Coronal view showing the extensive tumor thrombus in the inferior vena cava (large arrow) and the left ovarian vein (small arrows). **(F)** Coronal view from magnetic resonance venography (MRV) confirming the extensive filling defects in the inferior vena cava and right ventricle (arrows).

Imaging studies, including CT and MRI, demonstrated a solid uterine mass measuring 8 × 6.5 cm, with involvement of both adnexa. A 2.5 × 1.3 cm nodule was identified in the left lateral segment of the liver. Multiple tumor thrombi were present in the bilateral common iliac veins, left external and internal iliac veins, inferior vena cava, right atrium, right ventricle, main pulmonary artery, and branches of the pulmonary arteries (Figure 1). No gross visceral abnormalities were observed.

Echocardiography revealed a solid mass with moderate to low echogenicity in the inferior vena cava, extending into the right atrium. The mass was irregular in shape, measuring approximately 4.9 × 2.68 cm, and exhibited significant mobility, traversing the tricuspid valve into the right ventricle during the cardiac cycle.

Fluorodeoxyglucose (^18^F-FDG) PET-CT demonstrated abnormally increased radiotracer uptake in the right atrium, right ventricle, and main pulmonary artery, with a maximum standardized uptake value (SUVmax) of 2.25. Additionally, a slightly hypoattenuating nodule in the left lateral segment of the liver showed mild radiotracer uptake (SUVmax = 2.48). A large soft tissue mass in the lower abdomen and pelvis measured approximately 9.9 × 8.5 cm, with an SUVmax of 3.91. Multiple foci of abnormally increased radiotracer uptake were observed within the inferior vena cava, left renal vein, bilateral common iliac veins, and left external and internal iliac veins, with an SUVmax of 3.59. Both adnexa and the lower segment of the left ureter were involved, with accompanying hydronephrosis and hydroureter on the left side.

Given the extensive tumor involvement, including the heart and major vascular structures, the patient was at imminent risk of acute heart failure, pulmonary embolism, and sudden death. Consequently, the multidisciplinary team (MDT), comprising specialists in cardiothoracic surgery, gynecology, hepatobiliary surgery, urology, medical oncology, radiology, nuclear medicine, anesthesiology, and ICU management, was consulted. Due to the patient's critical condition and the extensive tumor thrombus, adjuvant endocrine therapy and chemotherapy were deemed contraindicated. The MDT decided to proceed with extensive open surgery aimed at complete tumor resection.

### 2.3 Surgical procedure

According to the 2023 International Federation of Gynecology and Obstetrics (FIGO) staging criteria and treatment recommendations for uterine sarcoma (17, 18), the patient in this case was classified as stage IVB. The surgery was conducted in a staged approach with the gynecology and cardiothoracic teams collaborating.

The patient was placed under general anesthesia, the gynecological team began the procedure by performing a cytoreductive surgery for the uterine tumor, including total hysterectomy, bilateral salpingo-oophorectomy, resection of the left ovarian ligament, and para-aortic lymph node dissection. Intraoperatively, a massive abdominal tumor was found, compressing the left ureter, necessitating the placement of a stent via transurethral ureteroscopy. The uterus was enlarged, resembling a 6-month pregnancy, with an uneven surface. A firm, palpable tumor was identified within the left ovarian ligament, extending to the left renal vein via the uterine angle. A large, fixed, hard mass was present on the left side of the uterus, involving the left pelvic wall, left ureter, and left iliac vessels.

The cardiothoracic surgery team then performed a right atriotomy. Cardiopulmonary bypass (CPB) was established through cannulation of the ascending aorta, right iliac vein, and superior vena cava. A median sternotomy was performed, followed by opening the pericardium. After systemic heparinization, CPB was initiated, a right atriotomy was performed, and revealing a cord-like tumor extending from the right atrium into the right ventricle and pulmonary artery. The tumor was well-defined, firm, smooth in texture, and minimally adherent to the vessel walls. The tumor was transected at the IVC inlet, and complete *en bloc* resection of the intracardiac, pulmonary arterial, and IVC tumor components was achieved via the right atrial approach. A venous cannula was positioned in the right atrium, and both superior and inferior vena cavae were temporarily occluded. The IVC was incised proximal to the right iliac vein, allowing complete retrograde extraction of the tumor thrombus. However, the tumor thrombus in the left iliac vein showed dense adherence to the vascular wall, requiring partial dissection followed by transection. The tumor thrombus in the left renal vein was completely removed proximally, and ligation and closure of the left renal vein were performed. After ensuring stable CPB, the machine was stopped, and transesophageal echocardiography confirmed no residual tumor in the heart or pulmonary arteries.

### 2.4 Pathological findings

**Figure 2:**
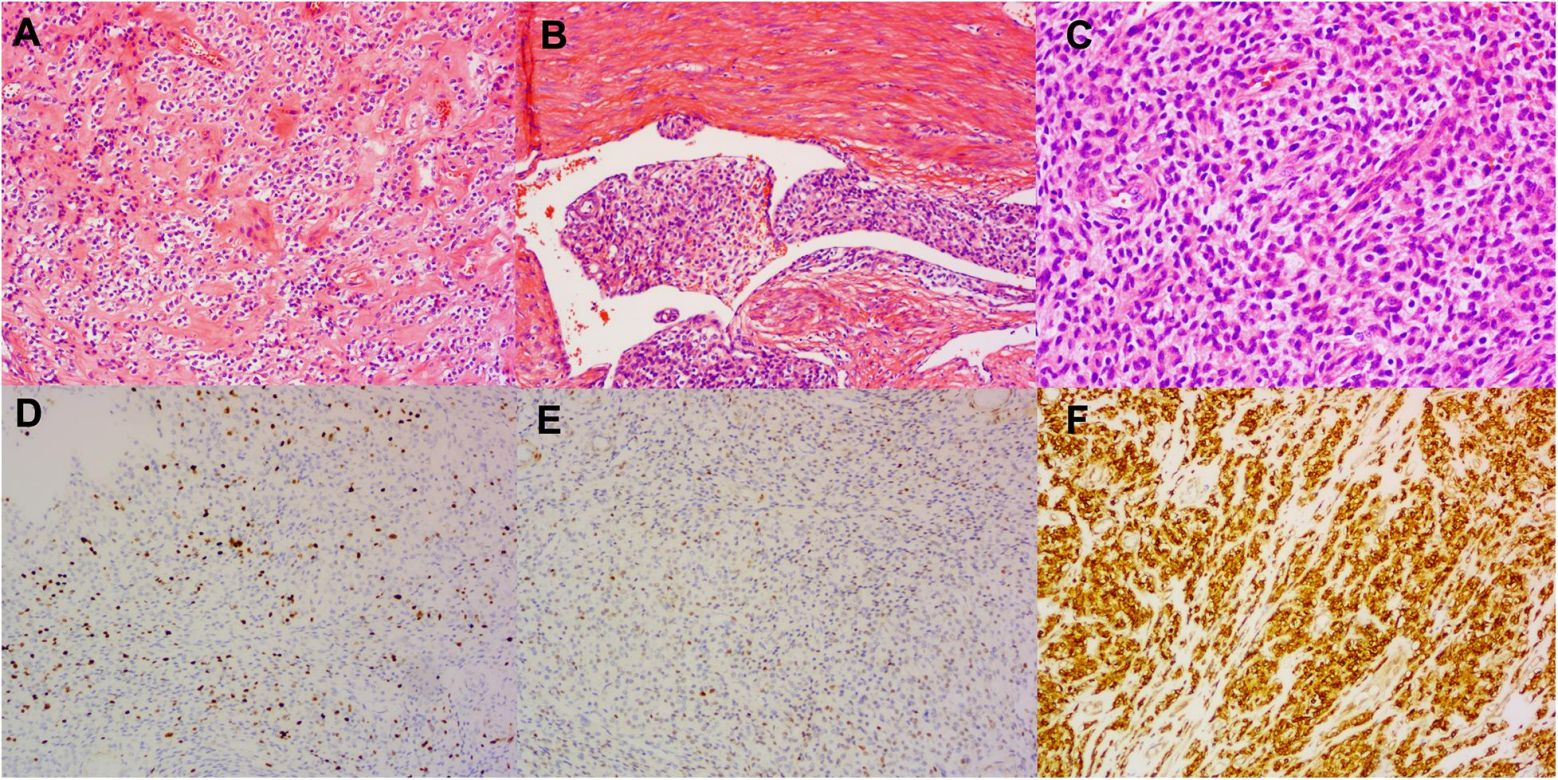
Pathological findings. **(A)** H&E staining (100 × magnification) showing uterine infiltration of the tumor. **(B)** H&E staining (100 ×  magnification) demonstrating intravascular tumor thrombus. **(C)** H&E staining (200 × magnification) showing the cellular morphology. **(D–F)** Immunohistochemical staining for Ki-67 **(D)**, Cyclin D1 **(E)**, and CD10 **(F)**, respectively.

Postoperative pathological examination confirmed the diagnosis of low-grade endometrial stromal sarcoma with extensive lymphovascular space invasion (Figure 2). Immunohistochemistry results revealed that the tumor was positive for CD10, estrogen receptor (ER), progesterone receptor (PR), CD31, and CD34, while it was negative for CK-PAN, EMA, *α*-SMA, Desmin, Caldesmon, WT1, Calretinin, Inhibin *α*, SF1 and CD117. The Ki-67 proliferation index was 10%, and p53 was wild-type. No metastasis was observed in the para-aortic or presacral lymph nodes.

### 2.5 Postoperative course and outcome

**Figure 3:**
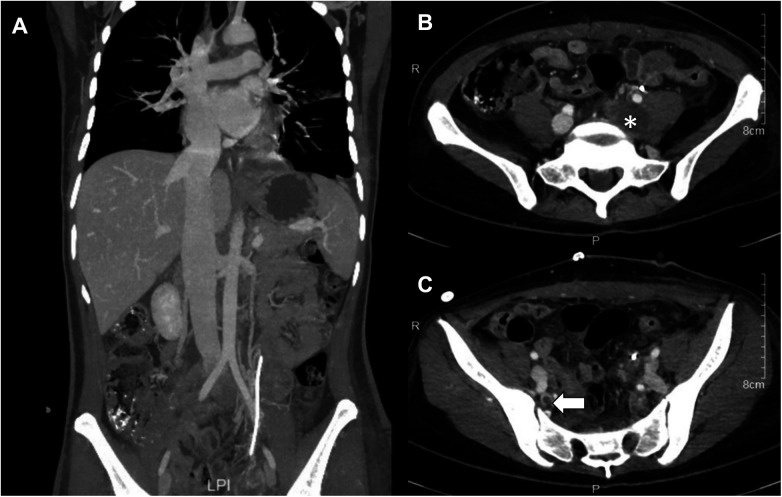
Postoperative computed tomography venography (CTV) at 6-day follow-up. **(A)** Coronal view demonstrating the complete resolution of the tumor thrombus in the right atrium, right ventricle, left ovarian vein, and inferior vena cava. **(B)** Axial view showing residual tumor thrombus in the left common iliac vein (asterisk). **(C)** Axial view showing residual tumor thrombus in the right internal iliac vein (arrow).

The patient had an uneventful recovery and was discharged on postoperative day 14. Follow-up CT on postoperative day 6 showed multiple filling defects in the left common iliac vein, left internal and external iliac veins, and right internal iliac vein, with no significant filling defects in the IVC, right atrium, right ventricle, or pulmonary artery (Figure 3). At the two-month postoperative follow-up, ultrasound revealed no obvious masses in the pelvis, and no filling defects were observed in the iliac veins. The spontaneous resolution of these filling defects on follow-up imaging strongly supports the interpretation that the initial postoperative findings represented bland venous thrombi related to surgical manipulation, rather than residual neoplastic tissue. The patient commenced postoperative adjuvant endocrine therapy with Letrozole (2.5 mg, once daily), which she continues to take to date and made a full recovery, resuming normal activities of daily living.

## 3 Discussion

Endometrial stromal sarcoma (ESS) accounts for 0.2% to 1% of all uterine malignancies and less than 10% of uterine sarcomas, making it the second most common uterine mesenchymal malignancy (9, 19). ESS can be classified into low-grade and high-grade tumors based on mitotic activity. Low-grade tumors display fewer than 10 mitotic figures per 10 high-power fields and have a 5-year survival rate ranging from 80% to 100%, with a recurrence rate of approximately 50%. In contrast, high-grade ESS has a 5-year survival rate of 25% to 55%, with a recurrence rate of about 33% (1, 2, 20). The clinical presentation of ESS is often nonspecific, with the most common symptoms being abnormal uterine bleeding. Other symptoms, such as abdominal pain, uterine enlargement, and dysmenorrhea, may also occur, though some patients may remain asymptomatic (4). Preoperative diagnosis of ESS is challenging due to the nonspecific nature of its clinical presentation, which overlaps with other uterine malignancies. At the time of initial diagnosis, the tumor may involve the parametrium and often metastasizes to pelvic lymph nodes via lymphatic channels and ovarian veins. However, involvement of major vessels or the heart is exceedingly rare, with only a few case reports documented (4, 7, 9–11). We hypothesize two potential mechanisms for ESS metastasis to the heart: (1) hematogenous spread of ESS to the lungs, followed by embolization to the right heart via the pulmonary veins (12, 20), and (2) direct extension of tumor thrombus via the venous system, particularly from the iliac veins or ovarian veins into the heart through the inferior vena cava (IVC). Most reported cases, including our own, support the latter mechanism (11, 21–23). This pattern of invasion is analogous to that seen in intravenous leiomyomatosis. The summary of the previously published cases with histologically proven LGESS and intracardiac extension were presented in Table 1.

**Table 1:** Published cases of LGESS with intracardiac extension.

Author (year)	Age	Cardiac extent	Surgical strategy	Additive treatment	Follow-up (outcome)
Yokoyama et al. (2004) (4)	48	RV	One-stage (Open heart + partial DHCA)	Chemotherapy (Ifosfamide, Epirubicin, Cisplatin)	Alive (Symptom-free)
Coganow et al. (2006) (25)	47	RA → RV → PA	One-stage (Sternotomy, CPB, beating heart)	Not specified	Alive (9 mo)
Renzulli et al. (2009) (22)	47	RA	One-stage (Sternotomy, CPB)	Radiotherapy + Tamoxifen	Alive (4.5 yr)
Scher et al. (2015) (10)	55	RA → PA	One-stage (Sternotomy, CPB, DHCA) after failed AngioVac	Anastrozole (Aromatase Inhibitor)	Alive (1 yr)
Zhang et al. (2015) (30)	45	RA → RV	Two-stage (Hysterectomy first; Sternotomy + CPB 25 days later)	Toremifene Citrate recommended	Alive (3 mo)
Nogami et al. (2016) (21)	58	RA	One-stage (Emergency Sternotomy, CPB)	Medroxyprogesterone Acetate (MPA)	Alive (1 yr)
Tadic et al. (2020) (26)	61	RA → RV	Surgery Ruled Out (High risk/Infiltration)	Hormonal + Anticoagulation (Enoxaparin)	Alive with disease (6 mo)
Chen et al. (2022) (23) Case 5	43	RA → RV	One-stage (TH + BSO + Thrombectomy)	None	Alive (20 mo)
Chen et al. (2022) (23) Case 7	46	RV	One-stage (TH + BSO + Thrombectomy)	Letrozole	Alive (6 mo)
Ge et al. (2024) (11)	48	RA	Surgical Intervention (Details not specified)	Not specified	Alive (Post-op recovery favorable)
Present Case	37	RA → RV → PA	One-stage (Sternotomy, CPB)	Letrozole	Alive

RA, right atrium; RV, right ventricle; PA, pulmonary artery; CPB, cardiopulmonary bypass; DHCA, deep hypothermic circulatory arrest; TH + BSO, Total Hysterectomy + Bilateral Salpingo-Oophorectomy.

Preoperative imaging plays a pivotal role in delineating the extent of LGESS, particularly when large pelvic masses are present. While standard imaging modalities like CT and MRI are essential for assessing tumor size and local involvement, they may struggle to detect fully mobile vascular and intracardiac tumors. Previous reports have highlighted instances where such tumors were mislocated on contrast-enhanced CT, emphasizing the limitations of these imaging techniques (21). Given these challenges, multimodal imaging-including ultrasound, enhanced CT, MRI, and PET—offers a more comprehensive assessment, albeit at a higher cost and increased time investment. Emerging evidence underscores the critical value of molecular imaging in this context. For instance, FDG PET/CT has proven indispensable for whole-body assessment, preventing the omission of rare metastatic sites such as the ischium or other occult lesions that standard anatomical imaging might miss (5). Similarly, SPECT/CT has demonstrated high diagnostic utility in detecting rare skeletal metastases in gynecological malignancies, further reducing the risk of under-staging (6). In our case, preoperative imaging identified tumor thrombi involving the bilateral common iliac veins, IVC, left renal vein, right atrium, right ventricle, main pulmonary artery, and the proximal branches of both the left and right pulmonary arteries. Distinguishing between tumor thrombus and blood clots is a key diagnostic challenge, particularly with filling defects in the heart and major vessels. Blood clots can often be managed with anticoagulation to reduce their size and facilitate surgery, whereas tumor thrombus requires more aggressive treatment, typically involving chemotherapy or endocrine therapy. Accurate preoperative differentiation is crucial, as interventional treatments may fail in cases of extensive tumor thrombus (10). Building upon these considerations, imaging studies like PET/CT can provide valuable insights into the metabolic activity of filling defects, offering a better indication of whether these are tumor thrombi. In our patient, PET/CT revealed significantly increased SUV, consistent with high metabolic activity, which strongly suggested tumor thrombus over benign blood clots. These findings are consistent with previous literature (8), underscoring the utility of multimodal imaging in distinguishing between different types of thrombus and guiding clinical decision-making. In addition to its role in diagnosing vascular involvement, preoperative imaging is crucial for evaluating tumor compression, particularly of the ureters. Tumor-related compression can lead to hydronephrosis, which, if left untreated, may result in renal failure. In our case, compression of the left ureter and hydronephrosis were evident, accompanied by tumor thrombus in the left renal vein. However, the patient fortunately exhibited no significant renal function abnormalities at the time of presentation. These findings underscore the importance of early recognition and proactive management of such complications. Ultimately, the complex interplay of tumor thrombus, vessel involvement, and the potential for metastasis requires a thorough and accurate preoperative assessment. Our case emphasizes the importance of maintaining clinical vigilance for unusual vascular invasion by LGESS, especially when dealing with advanced disease or involvement of major vessels and the heart.

Regarding surgical management, total hysterectomy with bilateral salpingo-oophorectomy remains the standard treatment for early-stage ESS (stage I-II). Although the preservation of ovaries in young patients with stage I-II disease is a consideration for fertility preservation, it may lead to a higher recurrence rate. Thus, oophorectomy is generally recommended for patients who do not desire fertility preservation (1, 24). For patients with stage I-II disease who wish to preserve fertility, ovarian conservation should be approached cautiously after a thorough discussion regarding the potential increased risk of recurrence, with oophorectomy recommended after childbearing is complete. While LGESS typically spreads via lymphatic or hematogenous routes to the lungs and pelvis, intracardiac metastasis represents a distinct and rare entity characterized by a “creeping” intravenous growth pattern. This behavior mimics intravenous leiomyomatosis, where the tumor extends from the pelvic veins into the IVC and subsequently into the right atrium, rather than manifesting as discrete hematogenous metastases. As illustrated in Table 1, the extent of cardiac involvement varies significantly, ranging from isolated right atrial masses to extensive propagation into the right ventricle and pulmonary arteries. The surgical management of these complex cases has evolved toward a coordinated multidisciplinary approach (22). Historical reviews (2) have noted the use of both two-stage and one-stage procedures; however, contemporary literature strongly favors a one-stage radical resection (4, 10, 21, 22, 25). The majority of recent successful cases (10, 23, 25), utilized a single-stage sternotomy and laparotomy with CPB. This approach minimizes the risks of tumor embolization and hemodynamic instability that may occur between staged procedures. While deep hypothermic circulatory arrest (DHCA) (10) was required in select cases to ensure a bloodless field for extensive caval resection, other authors (4, 23, 26) have successfully performed thrombectomy using CPB on a beating heart or even without CPB under close transesophageal echocardiographic monitoring when the tumor was non-adherent. In our case, the use of CPB allowed for the safe and complete *en bloc* resection of the extensive intracardiac and pulmonary thrombus, consistent with the successful outcomes observed in the literature. Despite the dramatic presentation of a “heart tumor,” the prognosis for intracardiac LGESS appears favorable when complete resection is achieved. In contrast to high-grade sarcomas, patients with LGESS often experience prolonged disease-free survival. Multiple patients have achieved disease-free intervals exceeding one year, with some surviving up to 4.5 years post-operatively (22). Adjuvant therapy plays a critical role in this long-term management. Hormonal suppression (26), particularly with aromatase inhibitors like letrozole or anastrozole (10, 23), has been widely adopted to reduce the risk of recurrence in this estrogen-dependent malignancy. The favorable outcomes documented in Table 1 support the decision for aggressive surgical intervention, even in the presence of massive vascular tumor burden, as it offers the best chance for long-term survival and symptom resolution. Our findings are consistent with the largest retrospective series to date by Chen et al. (23), who reported eight cases of LGESS with intravenous extension. It is noteworthy that while their series represents a significant volume, only two of the eight patients exhibited intracardiac involvement (extending to the right atrium or ventricle), with the remainder confined to the inferior vena cava and iliac veins. In contrast, our patient presented with a more extensive tumor burden that propagated further into the pulmonary arteries, a presentation seen in only a minority of reported cases. Despite the increased surgical complexity required for our patient, our favorable short-term outcome mirrors that of the Chen et al. cohort, where complete resection yielded a 100% disease-free survival rate over a mean follow-up of 34.5 months. Together, these data reinforce the conclusion that aggressive, multidisciplinary surgical management is safe and effective, even for the most extensive variants of this disease.

Post-operative imaging presented a complex picture. An initial CT venogram on day 6 revealed a filling defect in the iliac vessel, which could not definitively exclude residual tumor. However, subsequent ultrasound surveillance at 6 months demonstrated complete patency of the vessel, suggesting the initial finding was likely related to post-surgical thrombus which had since resolved. The patient remains without clinical or sonographic evidence of recurrence. Our case supports the notion that single-stage surgery, when carefully planned and executed with the support of a multidisciplinary team, can be an effective treatment for advanced metastatic ESS. In advanced LGESS cases, radical surgery remains justified, with the potential to improve recurrence-free survival. Other treatment options include endocrine therapy, which has shown promise in the management of recurrent or metastatic LGESS. Hormonal therapies, such as progestins, aromatase inhibitors, and gonadotropin-releasing hormone analogs, have been shown to be effective in some patients. Studies indicate that hormone therapy is associated with improved survival outcomes compared to observation (9, 26, 27). For patients who are not candidates for surgery or have advanced or recurrent diseases, chemotherapy can be considered. Recommended agents include doxorubicin, docetaxel, and ifosfamide (27). In patients with heart and large vessel thrombi who decline surgery, chronic anticoagulation therapy is a viable option, as both tumor and non-tumor thrombi may coexist in these cases (26). Previous studies have suggested that adjuvant radiotherapy can help reduce the local recurrence rate in patients who are not amenable to surgery (12).

The prognosis of LGESS with metastasis is largely dependent on the extent of metastasis and the success of surgical resection. While the 5-year survival rate for low-grade tumors is typically favorable, the recurrence rate remains high, especially when metastasis involves vital organs such as the heart (1, 20). In our patient, despite the extensive metastasis to both the IVC and right atrium, the single-stage surgical resection achieved a favorable outcome, suggesting that aggressive surgical management can improve recurrence-free survival in advanced cases. Given the high recurrence rate and potential for further metastasis, long-term follow-up with imaging and clinical monitoring is crucial to assess for recurrence and manage any complications.

A notable limitation of the present study is the absence of molecular genetic analysis. LGESS is frequently associated with specific chromosomal translocations, most notably the JAZF1-SUZ12 fusion, which is detected in approximately 50% of cases (28). Other recurrent alterations include *PHF1* rearrangements (e.g., *JAZF1-PHF1*, *EPC1-PHF1*). While our diagnosis was robustly confirmed through characteristic histological features and a distinct immunohistochemical profile (CD10+, ER+, PR+), molecular markers are increasingly recognized for their diagnostic utility in equivocal cases. In rare presentations involving aggressive cardiac metastasis, molecular profiling could provide valuable insights into the biological drivers of such behavior. Future cases would benefit from molecular profiling to further characterize the disease biology and help identify potential therapeutic targets. Recent multicenter analyses have further elucidated the prognostic landscape of this disease. Borella et al. (29) demonstrated that while LGESS is typically indolent, it carries a persistent risk of late recurrence, reinforcing the critical value of hormone receptor assessment in guiding long-term adjuvant therapy. Their findings suggest that even in advanced stages, favorable outcomes can be achieved with appropriate surgical and adjuvant endocrine management. Integrating such comprehensive clinicopathological data with future molecular profiling will be essential for understanding the biological underpinnings of rare, aggressive phenotypes such as the intracardiac extension observed in our case.

## 4 Conclusion

This case underscores the importance of comprehensive preoperative assessment and multimodal imaging in evaluating the extent of tumor involvement in LGESS. Detailed imaging and a multidisciplinary approach facilitated a successful single-stage surgical resection. However, the patient's high risk for acute right heart failure, pulmonary embolism, and sudden death limited the use of preoperative anticoagulation or chemotherapy, preventing evaluation of tumor thrombus response and achieving R0 resection. Furthermore, genetic characteristics, including mutations such as JAZF1/SUZ12, JAZF1-PHF1, and EPC1-PHF1 fusions, remain insufficiently explored. The short follow-up period hinders assessment of long-term survival, particularly the 5-year survival rate. While cardiac and large vessel metastases in LGESS are rare, active treatment can significantly improve outcomes. Future research should focus on optimizing treatment strategies and evaluating long-term survival in advanced cases with vascular and cardiac involvement. Given the indolent nature of LGESS and its propensity for late recurrence, prolonged surveillance extending beyond one decade is required to confirm the durability of these surgical outcomes.

## Data Availability

The original contributions presented in the study are included in the article/Supplementary Material, further inquiries can be directed to the corresponding author.

## References

[1] Yoon A Park JY Park JY Lee YY Kim TJ Choi CH Prognostic factors and outcomes in endometrial stromal sarcoma with the 2009 FIGO staging system: a multicenter review of 114 cases. Gynecol Oncol. (2014) 132(1):70–5. 10.1016/j.ygyno.2013.10.029 24184602

[2] Thiel FC Halmen S. Low-Grade endometrial stromal sarcoma - a review. Oncol Res Treat. (2018) 41(11):687–92. 10.1159/000494225 30317238

[3] Nathan D Szeto W Gutsche J Min H Kalapatapu V. Metastatic endometrial sarcoma with Inferior vena caval and cardiac involvement: a combined surgical approach. Vasc Endovascular Surg. (2014) 48(3):267–70. 10.1177/1538574413518118 24399127

[4] Yokoyama Y Ono Y Sakamoto T Fukuda I Mizunuma H. Asymptomatic intracardiac metastasis from a low-grade endometrial stromal sarcoma with successful surgical resection. Gynecol Oncol. (2004) 92(3):999–1001. 10.1016/j.ygyno.2003.11.049 14984976

[5] Katal S Al-Ibraheem A Abuhijla F Abdlkadir A Eibschutz L Gholamrezanezhad A. Correlative imaging of the female reproductive system. In: Gholamrezanezhad A Assadi M Jadvar H, editors. Radiology-Nuclear Medicine Diagnostic Imaging. Hoboken, NJ: John Wiley & Sons, Ltd. (2023). p. 554–76. Available online at: https://onlinelibrary.wiley.com/doi/abs/10.1002/9781119603627.ch20 (Accessed December 4, 2025).

[6] Al-Ibraheem A Abdlkadir AS. The outcome of progressive uterine sarcoma with potential bone involvement. World J Nucl Med. (2023) 22(1):48–51. 10.1055/s-0042-1757285 36923970 PMC10010860

[7] Wood CL Sederberg J Russ P Seres T. Cystic appearance of low-grade endometrial stromal sarcoma in the right atrium: case report. Cardiovasc Ultrasound. (2011) 9(1):23. 10.1186/1476-7120-9-23 21864385 PMC3184038

[8] Luo Y Feng R Li F. FDG PET/CT appearance of tumor thrombus of ovarian vessels masquerading as retroperitoneal fibrosis. Clin Nucl Med. (2015) 40(6):501–3. 10.1097/RLU.0000000000000655 25546209

[9] Manuel V Dinato FJ Gutierrez PS Siqueira SAC Gaiotto FA Jatene FB. Cardiac metastatic endometrial stromal sarcoma 17 years after hysterectomy. J Card Surg. (2017) 32(10):636–8. 10.1111/jocs.13221 28967156

[10] Scher D Nghiem W Aziz S Rahbar R Banks W Venbrux A Endometrial stromal sarcoma metastatic from the uterus to the Inferior vena Cava and right atrium. Tex Heart Inst J. (2015) 42(6):558–60. 10.14503/THIJ-14-4235 26664311 PMC4665285

[11] Ge Z Yang X Li J. Endometrial stromal sarcoma with intracardiac extension through inferior vena cava. Eur Heart J. (2024) 45(34):3186. 10.1093/eurheartj/ehae391 38953792

[12] Shakerian B Mandegar M Moradi B Roshanali F. Heart and lung metastases from endometrial stromal sarcoma in a forty-two-year-old woman. Res Cardiovasc Med. (2015) 4(3):8. 10.5812/cardiovascmed.26066v2 PMC4588706 26436070

[13] Phillips MR Bower TC Orszulak TA Hartmann LC. Intracardiac extension of an intracaval sarcoma of endometrial origin. Ann Thorac Surg. (1995) 59(3):742–4. 10.1016/0003-4975(94)00580-X 7887724

[14] Abrams J Talcott J Corson JM. Pulmonary metastases in patients with low-grade endometrial stromal sarcoma. Clinicopathologic findings with immunohistochemical characterization. Am J Surg Pathol. (1989) 13(2):133–40. 10.1097/00000478-198902000-00006 2916727

[15] Gnangnon FHR Lawani I Natta N’tcha HN Haag EK Dossou FM Mehinto DK. A giant metastatic low-grade endometrial sarcoma requiring surgical management. Int J Surg Case Rep. (2022) 94:107163. 10.1016/j.ijscr.2022.107163 35658315 PMC9092967

[16] Devins KM Mendoza RP Shahi M Ghioni M Alwaqfi R Croce S Low-Grade endometrial stromal sarcoma: clinicopathologic and prognostic features in a cohort of 102 tumors. Am J Surg Pathol. (2025) 49(10):977–91. 10.1097/PAS.0000000000002428 40434060

[17] Berek JS Matias-Guiu X Creutzberg C Fotopoulou C Gaffney D Kehoe S FIGO Staging of endometrial cancer: 2023. Int J Gynaecol Obstet. (2023) 162(2):383–94. 10.1002/ijgo.14923 37337978

[18] Matsuo K Klar M Song BB Roman LD Wright JD. Validation of the 2023 FIGO staging schema for advanced endometrial cancer. Eur J Cancer. (2023) 193:113316. 10.1016/j.ejca.2023.113316 37741790

[19] Cui R Yuan F Wang Y Li X Zhang Z Bai H. Clinicopathological characteristics and treatment strategies for patients with low-grade endometrial stromal sarcoma. Medicine (Baltimore). (2017) 96(15):e6584. 10.1097/MD.0000000000006584 28403089 PMC5403086

[20] Gadducci A Multinu F De Vitis LA Cosio S Carinelli S Aletti GD. Endometrial stromal tumors of the uterus: epidemiology, pathological and biological features, treatment options and clinical outcomes. Gynecol Oncol. (2023) 171:95–105. 10.1016/j.ygyno.2023.02.009 36842409

[21] Nogami Y Yamagami W Maki J Banno K Susumu N Tomita K Intravenous low-grade endometrial stromal sarcoma with intracardiac extension: a CASE OF inaccurate tumor location on contrast-enhanced computed tomography. Mol Clin Oncol. (2016) 4(2):179–82. 10.3892/mco.2015.691 26893856 PMC4734120

[22] Renzulli P Weimann R Barras JP Carrel TP Candinas D. Low-grade endometrial stromal sarcoma with inferior vena cava tumor thrombus and intracardiac extension: radical resection may improve recurrence free survival. Surg Oncol. (2009) 18(1):57–64. 10.1016/j.suronc.2008.07.003 18708288

[23] Chen J Wang J Cao D Yang J Huang H Pan L Low-grade endometrial stromal sarcoma with intracaval or intracardiac extension: a retrospective study of eight cases. Arch Gynecol Obstet. (2022) 306(5):1799–806. 10.1007/s00404-021-06373-4 35094105

[24] Yano Y Yamasaki Y Yamanaka K Nishimoto M Nagamata S Terai Y. A case of a recurrent low-grade endometrial stromal sarcoma extending to the inferior vena cava (IVC) after the primary fertility-sparing surgery. Int J Surg Case Rep. (2023) 111:108857. 10.1016/j.ijscr.2023.108857 37741074 PMC10520521

[25] Coganow M Das BM Chen E Crestanello JA. Single-Stage resection of a mixed endometrial stromal sarcoma and smooth muscle tumor with intracardiac and pulmonary extension. Ann Thorac Surg. (2006) 82(4):1517–9. 10.1016/j.athoracsur.2006.02.038 16996971

[26] Tadic M Belyavskiy E Cuspidi C Pieske B Haßfeld S. Right heart masses in a patient with endometrial stromal sarcoma. J Clin Ultrasound. (2020) 48(2):117–20. 10.1002/jcu.22732 31074021

[27] Jibiki M Inoue Y Sugano N Iwai T Katou T. Tumor thrombectomy without bypass for low-grade malignant tumors extending into the Inferior vena Cava: report of two cases. Surg Today. (2006) 36(5):465–9. 10.1007/s00595-005-3175-4 16633754

[28] Hrzenjak A. JAZF1/SUZ12 Gene fusion in endometrial stromal sarcomas. Orphanet J Rare Dis. (2016) 11:15. 10.1186/s13023-016-0400-8 26879382 PMC4754953

[29] Borella F Bertero L Cassoni P Piovano E Gallio N Preti M Low-Grade uterine endometrial stromal sarcoma: prognostic analysis of clinico-pathological characteristics, surgical management, and adjuvant treatments. Experience from two referral centers. Front Oncol. (2022) 12:883344. 10.3389/fonc.2022.883344 35847944 PMC9280128

[30] Zhang AQ Xue M Wang DJ Nie WP Xu DB Guan XM. Two-stage resection of a disseminated mixed endometrial stromal sarcoma and smooth muscle tumor with intravascular and intracardiac extension. Taiwan J Obstet Gynecol. (2015) 54(6):776–9. 10.1016/j.tjog.2014.12.010 PMID: 26701002 26701002

